# Colorectal surgery in 2023: *BJS Open* highlights and editors’ choices

**DOI:** 10.1093/bjsopen/zrae006

**Published:** 2024-02-07

*BJS Open* is delighted to have published a wide selection of high-quality manuscripts across the breadth of surgery and colorectal surgery. In 2023 we have published papers covering colorectal topics from translational science to the latest techniques and patient-reported outcome measures and much more.

Starting with papers that have impacted cancer management, the first paper is a practice-changing paper using MRI to assess the sigmoid take off and to anatomically define sigmoid compared to rectal cancers^1^. In this large series, the authors demonstrated how this new classification could change the multidisciplinary management of rectal tumours. Of 1302 rectal cancers re-evaluated, 13.1% were reclassified as having sigmoid cancer. Of the 170 reclassified patients, 93 would have been offered a different neoadjuvant or adjuvant treatment according to the Dutch guidelines. This takes us on to the next editors’ choice assessing the variation in response to radiotherapy for rectal cancer. Sulit *et al.* explored human gene and microbial biomarkers in a cohort of rectal cancer patients who underwent chemoradiotherapy^2^. In this small cohort they demonstrated that complete responders have different immune responses, including immunoglobulin production, B-cell activation, and response to bacteria. They also identified an increased abundance of *Ruminococcaceae* bacteria and *Bacteroides thetaiotaomicron*. We look forward to receiving more research in the field of microbiomics and investigating the influence of micro-organisms of health and disease.

There is debate on the optimal time to adjuvant chemotherapy to achieve the balance of improving long-term outcomes while reducing adverse effects. An RCT recruited 443 patients randomized to early (10–14 days) *versus* conventional (24–28 days) chemotherapy^3^. This demonstrated no difference in toxicity, surgical complications, and quality of life, and suggests that starting chemotherapy within 2 weeks is safe.

Another area of coloproctology that divides opinion is the use of mechanical bowel preparation and preoperative oral and intravenous antibiotics. A network meta-analysis comprising 60 RCTs and 16 314 patients^4^ aimed to determine the optimal bowel preparation strategy. The authors concluded that oral and intravenous antibiotics with or without mechanical bowel preparation reduced surgical site infection and anastomotic leaks and should be the standard of care.

Moving on to emergency surgery, appendicitis is one of the most common presentations in emergency general surgery and there has been increasing research into non-operative management, particularly during the COVID-19 pandemic. The CASCADE study was a prospective multicentre observational study of 1464 children treated for uncomplicated appendicitis at 74 hospitals in the UK and Ireland^5^. Thirty per cent of patients underwent non-operative management and the overall success rate of non-operative management at 1 year was 63.1%. This study suggests that non-operative management of simple appendicitis is an option for selected patients.

Another challenge for the general surgeon is adhesional small bowel with the main options between early operative management and a trial of conservative management. Mortensen and co-authors investigated 201 patients who were prospectively recruited from six acute hospitals in Denmark and compared operative with non-operative management^6^. Patients undergoing operative treatment had significantly better 1-year recurrence-free survival compared with patients managed non-operatively (operative 92.5% *versus* non-operative 66.6%, *P* < 0.001). However, patients with non-operative management had a shorter length of stay. Multivariable analysis demonstrated that operative treatment was associated with decreased risk of recurrence (HR 0.22 (95% c.i. 0.10 to 0.48), *P* < 0.001) but an increased all-cause mortality rate (HR 2.48 (95% c.i. 1.13 to 5.46), *P* = 0.024).

The final editors’ choice is an under-represented area of research on the impact of defunctioning loop ileostomy as part of anterior resection. Rutegård *et al.* analysed 5355 patients and at 5 years follow-up 7.2% of defunctioned patients developed acute or chronic renal failure compared to 3.3% in the non-stoma group^7^. This suggests that there is a need for more research into better fluid management, stoma types, or stoma avoidance strategies.

The editors would like to thank all authors, peer reviewers and readers and we look forward to receiving your contributions and collaborating with you all in 2024!
